# *PCK1* and *SLC22A2* gene variants associated with response to metformin treatment in type 2 diabetes

**DOI:** 10.1371/journal.pone.0305511

**Published:** 2025-02-10

**Authors:** Sophie St-Amour, Laurence Tessier, Janie Harnois, Catherine Allard, Alexandre Lavoie, Philippe Caron, Luigi Bouchard, Patrice Perron, Karine Tremblay

**Affiliations:** 1 Research Center, Centre intégré universitaire de santé et de services sociaux du Saguenay–Lac-Saint-Jean, Saguenay, Quebec, Canada; 2 Department of Pharmacology-physiology, Faculty of Medicine and Health Sciences, Université de Sherbrooke, Saguenay, Quebec, Canada; 3 Research Center, Centre hospitalier universitaire de Sherbrooke, Université de Sherbrooke, Quebec, Canada; 4 Unité de Recherche Clinique et Epidemiologique, Centre de Recherche du Centre Hospitalier Universitaire de Sherbrooke, Sherbrooke, Quebec, Canada; 5 Pharmacy Department, Centre intégré universitaire de santé et services sociaux du Saguenay-Lac-Saint-Jean, Saguenay, Quebec, Canada; 6 Endocrinology Division, Centre intégré universitaire de santé et de services sociaux du Saguenay–Lac-Saint-Jean, Saguenay, Quebec, Canada; 7 Department of Biochemistry and Functional Genomics, Faculty of Medicine and Health Sciences, Université de Sherbrooke, Saguenay, Quebec, Canada; 8 Department of Medical Biology, CIUSSS Saguenay-Lac-Saint-Jean, Saguenay, Quebec, Canada; 9 Department of Medicine, Faculty of Medicine and Health Sciences, Université de Sherbrooke, Saguenay, Quebec, Canada; Zhejiang University of Technology, CHINA

## Abstract

Type 2 diabetes (T2D) is a chronic disorder affecting 462 million worldwide, often managed with metformin as first-line treatment. However, metformin’s response varies among individuals, including up to 30% experiencing serious adverse drug reactions (ADRs) and 20-50% inefficacy. These differences may be due to various factors, including pharmacogenetic (PGx) variants. The PGx variants documented so far could affect both the safety and efficacy of metformin, but due to a lack of replication studies, none reached the clinical evidence-level needed to be used as a predictive marker for treatment response. Therefore, this study aims to evaluate the association between the presence of candidate PGx variants and metformin response in T2D subjects. We conducted an association study involving 108 T2D participants currently or previously treated with metformin. A characterization of their therapeutic response was carried out through questionnaires and pharmacological profile reviews. DNA samples were collected during their single visit to perform genotyping of 24 selected candidate PGx variants. Association analyses between candidate PGx variants and metformin response were performed. Among the subjects included in the analyses (n =  84), 25% were non-responders, and 58% experienced ADRs. At the time of study enrollment, 93.9% of non-responders continued to use metformin. The odds of being a non-responder to metformin are 5.6 times higher for homozygous carriers of the alternative allele of a variant within the *PCK1* gene (rs4810083) compared to the other genotypes (95% interval confidence [1.9–16.6]). Two variants in perfect linkage disequilibrium within the *SLC22A2* gene (rs316019 and rs316009) were associated with increase odds of having ADRs, where homozygous genotype carriers are 7.3 times more likely to have ADRs presentation (95% interval confidence [1.85–29.01]). This study identified associations between *PCK1* and *SLC22A2* candidate PGx variants and metformin response in T2D treatment. Additional genetic and functional studies are necessary to elucidate the variants’ impact in metformin’s pharmacological mechanisms.

## Introduction

Diabetes, the most common endocrine disorder, affected over 5.7 million individuals in Canada in 2022, including type 1 and type 2 [1], and is projected to affect 693 million people worldwide by 2045 [2]. This is a significant rise compared to earlier data, and diabetes continues to represent a major public health challenge in Canada. Diabetes costs approximately $30 billion to Canada’s healthcare system annually [1]. This chronic disease predisposes affected people to many complications including vascular and kidney diseases, retinopathy and neuropathy [3].

Type 2 diabetes (T2D) is the most common form of diabetes accounting for more than 90% of cases [4]. T2D is primarily caused by an increased peripheral insulin resistance accompanied by the pancreatic beta cells being unable to produce and secrete sufficient amounts of insulin to maintain blood glucose homeostasis [3]. Insulin production and secretion defect as well as resistance hinder glucose uptake and utilization by the effector cells, leading to hyperglycemia [3], a condition that can lead to the complications associated with diabetes [3,4]. Environmental and genetic factors appear to modulate the development of T2D [3,5]. In Canada, if HbA_1C_ exceeds the targeted threshold by more than 1.5% after three months of lifestyle modification, metformin is recommended immediately as first-line medication [6]. Metformin primarily reduces hepatic gluconeogenesis and, to a lesser extent, hepatic glycogenolysis, while increasing insulin-mediated glucose uptake in skeletal muscles and fat cells [7].

The global burden of diabetes continues to rise, affecting millions of individuals worldwide, placing immense strain on healthcare systems, and necessitating effective, individualized treatments [1,2]. A significant challenge in managing diabetes is the variability in patients’ responses to medications like metformin. It is well recognized that interindividual response to medication may vary, sometimes requiring weeks to optimize treatment and control symptoms sometimes leading to adverse drug reactions (ADRs). For instance, metformin users may experience ADRs such as nausea and diarrhea, which affect about 30% of users, with 5% discontinuing treatment due to severe reactions [7–10]. Metformin users also face a higher risk of vitamin B12 deficiency [8] and in rare cases, lactic acidosis occurs, representing the most severe and potentially fatal ADR [7]. On the other hand, some individuals may never achieve the expected therapeutic effect, the so-called non-responders (NR). Regarding metformin’s efficacy, 20% to 50% of users are reported as NR [11,12]. NRs are difficult to identify. Current clinical guidelines recommend the addition of a second-line drug without further investigation if glycemic targets are not achieved after three to six months [12–16]. In this context, identifying metformin NRs may take a few years [17]. This approach can lead to prolonged periods of ineffective treatment for NRs, while others may experience ADRs without the expected benefits. Identifying metformin NR or those at risk of ADRs prior to initiation could significantly impact treatment delay, glycemic control, costs and quality of life of people living with diabetes.

Drug response variability, which complicates diabetes management, arises from a complex combination of genetic and lifestyle factors [18–20], including age, concomitant therapies, drug interactions and genetic variants [18]. When assessing metformin response, factors such as body mass index (BMI), medication compliance and dosage account for only part of the response variability [21]. Another fraction of this variability may be due to the presence of pharmacogenetic (PGx) variants [21]. Although PGx application is in its infancy in T2D, at least 74 PGx variants (distributed in 40 known genes) have been documented to influence metformin efficacy or safety profile [22,23]. The hepatocytes transporters (*SLC22A1*, *SLC22A2*, *SLC47A1* or *SLC2A2*) and kinase proteins involved in gluconeogenesis (*ATM*) [22,23] were the only genes replicated in at least two independent studies. Other associated genes are involved in intracellular signaling (*PRKAA1/2, STK11*) or function as transcription factors (*SP1, PPARGC1A/B, HNF4A, TCF7L2* and *HNF1B*) [22–24]. Additionally, there are genes associated with energy homeostasis and metabolism (*PEPCK*, *GCK* and *CPA6*), as well as those involved in insulin signaling and sensitivity (*IRS1*, *KCNJ11*, *CSMD1*, *CDKN2A/B*, *CAPN10* and *NBEA*) [22,24–28].

In the present study, we aimed to assess the association between candidate PGx variants and metformin response phenotypes in T2D treatment. Our results support that candidate PGx variants should become clinical guideline annotations.

## Results

### Characteristics of the participants

S1 Table presents the participants’ characteristics related to health, lifestyle, phenotype, and ADRs. Briefly, 84 participants were included in the cohort of which, 25% (n =  21) were NRs and 59.0% (n =  49) have reported experiencing at least one ADR. All variables presented in S1 Table were tested for potential association with metformin efficacy and safety. Among all tested variables, seven were significantly associated with metformin’s efficacy: musculoskeletal diseases (p-value =  0.004), neurological diseases (p-value =  0.032), gastrointestinal diseases (p-value =  0.021), levels of HbA_1C_ at metformin initiation (p-value =  0.041), levels of HbA_1C_ and fasting glucose one year after metformin initiation (p-value =  < 0.0001) and taking another antidiabetic drug at study inclusion (p-value =  0.013). Additionally, four variables were significantly associated with metformin safety: diet score at T2D diagnosis (p-value =  0.033), diet score at study inclusion (p-value =  0.004), physical activity at study inclusion (p-value =  0.041) and mental health issues (p-value =  0.011). Detailed of the entire cohort’s characterisation is also available in  Supporting information.

### Genotype analysis and distribution in the cohort

A total of 34 variants were genotyped for all 105 participants who provided DNA samples (S2 Table). The Agena iPLEX technology was used at the Genome Québec “Centre d’expertises et de services” (CES) (Montréal, Canada) (platform genomequebec.com). Following genotyping, 7 single nucleotide variations (SNVs) were excluded due to their low call rates (<90%) and 3 SNVs due to the presence of only one genotype (no variation). Hardy-Weinberg Equilibrium (HWE) was tested for all variants, and all were found to be at equilibrium after Holm-Bonferroni correction. Two DNA samples were excluded from genetic analyses due to genotyping failures. Therefore, genetic statistical analyses were performed on a total of 82 participants for 24 SNVs (and only 80 participants with safety available and genotyping available) (S2 Table).

### Pharmacogenetic association with response to metformin

A comparison between responders and NRs was conducted for the selected variants selected for their reported effect on metformin efficacy (n =  14, S3 Table). Only *PCK1-*rs4810083 was significantly associated with metformin response in a recessive model (adjusted p-value =  0.0346; Table 1), but not in the dominant model (results presented in the S4 Table). NRs were predominantly homozygous for the alternative allele (55.0%). In comparison, in responders, only 18.0% were homozygous for the alternative allele. Thus, individuals that are homozygous carriers of the *PCK1-*rs4810083 alternative allele have 5.6 times more risk of being NRs to metformin than the 2 other genotype’s carriers (recessive model: OR =  5.56, IC 95% [1.86–16.63]).

**Table 1:** Genotype distribution between metformin’s responders and non-responders.

Genotypes *n (%)*	R (n = 61)	NR (n = 21)	p-value^a^	Adjusted p-value
*ABBC8* - rs4148609				
CC	28 (46.7)	8 (38.1)	0.678	1.000
CT	28 (46.7)	11 (52.4)		
TT	4 (6.7)	2 (9.5)		
*Recessive model* ^b^				
CC + CT	56 (93.3)	19 (90.5)	0.647	1.000
TT	4 (6.7)	2 (9.5)		
*Capn10* - rs3792269				
AA	43 (74.1)	15 (71.4)	0.834	1.000
AG	14 (24.1)	6 (28.6)		
GG	1 (1.7)	0 (0)		
*Recessive model*				
AA + AG	57 (98.3)	21 (100.0)	1.000	1.000
GG	1 (1.7)	0 (0)		
*CPA6* - rs2162145				
TT	3 (4.9)	0 (0)	0.819	1.000
TC	19 (31.1)	7 (33.3)		
CC	39 (63.9)	14 (66.7)		
*Recessive model*				
TT + TC	22 (36.1)	7 (33.3)	1.000	1.000
CC	39 (63.9)	14 (66.7)		
*CSMD1* - rs2954625				
CC	44 (74.6)	12 (60.0)	0.361	1.000
CT	13 (22.0)	7 (35.0)		
TT	2 (3.4)	1 (5.0)		
*Recessive model*				
CC + CT	57 (96.6)	19 (95.0)	1.000	1.000
TT	2 (3.4)	1 (5.0)		
*GCK* - rs2908289				
GG	43 (70.5)	17 (81.0)	0.761	1.000
GA	16 (26.2)	4 (19.0)		
AA	2 (3.3)	0 (0)		
*Recessive model*				
GG + GA	59 (96.7)	21 (100.0)	1.000	1.000
AA	2 (3.3)	0 (0)		
*HNF1B* - rs11868513				
GG	43 (70.5)	16 (76.2)	0.506	1.000
GA	15 (24.6)	3 (14.3)		
AA	3 (4.9)	2 (9.5)		
*Recessive model*				
GG + GA	58 (95.1)	19 (90.5)	0.598	1.000
AA	3 (4.9)	2 (9.5)		
*IRS1* - rs1801278				
CC	48 (78.7)	17 (81.0)	1.000	1.000
CT	12 (19.7)	4 (19.0)		
TT	1 (1.6)	0 (0)		
*Recessive model*				
CC + CT	60 (98.4)	21 (100.0)	1.000	1.000
TT	1 (1.6)	0 (0)		
*KCNJ11* - rs5219				
TT	14 (23.3)	5 (23.8)	0.690	1.000
CT	26 (43.3)	7 (33.3)		
CC	20 (33.3)	9 (42.9)		
*Recessive model*				
TT + CT	40 (66.7)	12 (57.1)	0.442	1.000
CC	20 (33.3)	9 (42.9)		
*KCNJ11* - rs7124355				
AA	13 (21.7)	5 (23.8)	0.685	1.000
AG	24 (40.0)	6 (28.6)		
GG	23 (38.3)	10 (47.6)		
*Recessive model*				
AA + AG	37 (61.7)	11 (52.4)	0.607	1.000
GG	23 (38.3)	10 (47.6)		
*NBEA* - rs57081354				
TT	53 (86.9)	20 (95.2)	0.435	1.000
TC	8 (13.1)	1 (4.8)		
CC	0 (0)	0 (0)		
*Recessive model*				
TT + TC	31 (100.0)	21 (100.0)	1.000	1.000
CC	0 (0)	0 (0)		
*PCK1* - rs4810083				
TT	15 (24.6)	2 (10.0) *^1^	**0.008**	0.104
TC	35 (57.4)	7 (35.0) *^2^		
CC	11 (18.0)	11 (55.0) *^1,2^		
*Recessive model*				
TT + TC	50 (82.0)	9 (45.0)	**0.003**	**0.039**
CC	11 (18.0)	11 (55.0)		
*PPARGC1A* - rs10213440				
TT	39 (65.0)	13 (65.0)	1.000	1.000
TC	19 (31.7)	7 (35.0)		
CC	2 (3.3)	0 (0)		
*Recessive model*				
TT + TC	58 (96.7)	20 (100.0)	1.000	1.000
CC	2 (3.3)	0 (0)		
*STK11* - rs741765				
CC	35 (58.3)	17 (81.0)	0.193	1.000
CT	23 (38.3)	4 (19.0)		
TT	2 (3.3)	0 (0)		
*Recessive model*				
CC + CT	58 (96.7)	21 (100.0)	1.000	1.000
TT	2 (3.3)	0 (0)		
*TCF7L2* - rs2908289				
CC	24 (40.0)	7 (35.0)	0.940	1.000
CT	26 (43.3)	10 (50.0)		
TT	10 (16.7)	3 (15.0)		
*Recessive model*				
CC + CT	50 (83.3)	17 (85.0)	1.000	1.000
TT	10 (16.7)	3 (15.0)		

Abbreviations: R, responders; NR, non-responders; *ABCC8,* ATP-binding cassette transporter sub-family C member 8; *CAPN10,* Calpain 10; *CPA6, C*arboxypeptidase A6; *CSMD1,* CUB and sushi multiple domains 1; *GCK,* Glucokinase; *HNF1B,* Hepatocyte nuclear factor 1; *IRS1,* Insulin receptor substrate 1; *KCNJ11,* Potassium inwardly rectifying channel subfamily J member 11; *NBEA,* Neurobeachin; *PCK1,* Phosphoenolpyruvate carboxykinase 1; *PPARGC1A,* Peroxisome proliferator-activated receptor gamma coactivator 1-alpha; *STK11,* Serine/threonine kinase 11; *TCF7L2,* Transcription factor 7-like 2.

^a^P-value of Fisher exact test comparing Rs to NRs. Bold numbers indicate significance (p < 0.05) and asterisks (*) indicate significance for *post-hoc* using Holm–Bonferroni adjustment.

^b^Dominant model results presented in supplement.

### Variants associated with safety to metformin

All PGx variants involved in metformin’s pharmacokinetic mechanisms (n =  9, S3 Table) were analyzed for association with ADRs occurrence using the three genetic models. *SLC22A2-*rs316019 was associated with presence of ADRs (codominant and recessive adjusted p-values =  0.024 and 0.024, respectively, Table 2). Dominant model couldn’t be tested because of the absence of homozygous for the reference allele in both safety groups (S5 Table). Among individuals without ADR, there were significantly more heterozygous (33.3%) than among those presenting with ADRs (6.4% of heterozygous) (p-value =  0.024). An odd ratio of 7.3 was found for homozygous carriers of *SLC22A2-*rs316019 alternative allele, which suggests a seven fold increase in odds of having ADRs (95% interval confidence [1.85–29.01]) when compared to individuals carrying a reference allele.

**Table 2:** Genotype distribution between metformin’s safety phenotypes in the studied cohort.

Genotypes *n (%)*	ADRs (n = 47)	No-ADR (n = 33)	p-value^a^	Adjusted p-value
*SLC22A1* - rs594709				
GG	6 (13.0)	2 (6.1)	0.637	1.000
GA	21 (45.7)	18 (54.5)		
AA	19 (41.3)	13 (39.4)		
*Recessive model* ^b^				
GG + GA	27 (58.7)	20 (30.6)	1.000	1.000
AA	19 (41.3)	13 (39.4)		
*SLC22A1* - rs1867351				
TT	25 (53.2)	18 (54.5)	1.000	1.000
TC	20 (42.6)	14 (42.4)		
CC	2 (4.3)	1 (3.0)		
*Recessive model*				
TT + TC	27 (58.7)	20 (30.6)	1.000	1.000
CC	19 (41.3)	13 (39.4)		
*SLC22A1* - rs12208357				
CC	44 (93.6)	30 (90.9)	0.687	1.000
CT	3 (6.4)	3 (9.1)		
TT	0 (0)	0 (0)		
*Recessive model*				
CC + CT	47 (100.0)	33 (100.0)	1.000	1.000
TT	0 (0.0)	0 (0.0)		
*SLC22A2* - rs316019/rs316009				
AA/TT	0 (0)	0 (0)	**0.003**	**0.024**
AC/TC	3 (6.4)	11 (33.3)		
CC/CC	44 (93.6)	22 (66.7)		
*Recessive model*				
AA/TT + AC/TC	3 (6.4)	11 (33.3)	**0.003**	**0.024**
CC/CC	44 (93.6)	22 (66.7)		
*SLC22A3* - rs2076828				
CC	10 (21.3)	16 (48.5) *	**0.033**	0.264
CG	30 (63.8)	13 (39.4) *		
GG	7 (14.9)	4 (12.1)		
*Recessive model*				
CC + CG	40 (85.1)	29 (87.9)	1.000	1.000
GG	7 (14.9)	4 (12.1)		
*SLC47A1* - rs8065082				
CC	21 (44.7)	12 (36.4)	0.485	1.000
CT	19 (40.4)	18 (54.5)		
TT	7 (14.9)	3 (9.1)		
*Recessive model*				
CC + CT	40 (85.1)	30 (90.9)	0.512	1.000
TT	7 (14.9)	3 (9.1)		
*SLC47A1* - rs2289669				
GG	24 (51.1)	13 (39.4)	0.389	1.000
GA	18 (38.3)	18 (54.5)		
AA	5 (10.6)	2 (6.1)		
*Recessive model*				
GG + GA	42 (90.9)	31 (93.9)	0.694	1.000
AA	5 (10.6)	2 (6.1)		
*SLC47A2* - rs12943590				
GG	23 (52.3)	15 (45.5)	0.682	1.000
GA	17 (38.6)	16 (48.5)		
AA	4 (9.1)	2 (6.1)		
*Recessive model*				
GG + GA	40 (90.9)	31 (93.9)	0.694	1.000
AA	4 (9.1)	2 (6.1)		
*SLC47A2* - rs34834489				
GG	19 (43.2)	9 (29.0)	0.180	1.000
GA	16 (36.4)	18 (58.1)		
AA	9 (20.5)	4 (12.9)		
*Recessive model*				
GG + GA	35 (79.5)	27 (87.1)	0.539	1.000
AA	9 (20.5)	4 (12.9)		

Abbreviations: ADR, adverse drug reaction; *SLC22A1,* Solute carrier family 22 member 1; *SLC22A2,* Solute carrier family 22 member 2; *SLC22A3,* Solute carrier family 22 member 3; *SLC47A1,* Solute carrier family 47 member 1; *SLC47A2,* Solute carrier family 47 member 2; *STK11,* Serine/threonine kinase 11; *TCF7L2,* Transcription factor 7-like 2.

^a^P-value of Fisher exact test comparing ADRs to No-ADR. Bold numbers indicate significance (p < 0.05) and asterisks (*) indicate significance for *post-hoc* using Holm–Bonferroni adjustment.

^b^Dominant model results presented in supplement.

## Discussion

Our study aimed to assess the association between previously associated PGx variants and the efficacy and safety of metformin as part of the treatment of T2D. A cohort of 84 participants was recruited and deeply phenotyped allowing us to find positive associations with *PCK1*-rs4810083 and *SLC22A2*-rs316019 and metformin response phenotypes.

Out of the 14 variants that were analyzed for metformin efficacy, *PCK1*-rs4810083 was associated with nonresponse in a recessive model. NRs were predominantly homozygous for the alternative allele (C), making them 5.6 times more likely to be an NR compared to those homozygous for the reference allele (T) or heterozygous. This variant was previously linked to metformin efficacy. Tkáč et al. did not find a significant association between the two alleles and metformin efficacy (HbA_1C_ <  7%) at 6 months [29]. This variant was also associated with metformin efficacy in the Diabetes Prevention Program where the allele (T) was associated with a lower diabetes incidence under metformin treatment which is in line with our findings [30].

The *PCK1* gene encodes the phosphoenolpyruvate carboxykinae 1 (PEPCK) enzyme, and the rs4810083 variant is located 2 kb upstream of this gene [31]. PEPCK is a crucial enzyme for gluconeogenesis, primarily occurring in the liver. It catalyzes the conversion of oxaloacetate into phosphoenolpyruvate while consuming a guanosine triphosphate [32]. Literature reports that *PCK1* can be underexpressed and overexpressed which results in different clinical conditions. For example, *PCK1* deficiency, although rare, manifests as hypoglycemia, lactic acidosis, liver failure, and excessive excretion of tricarboxylic acid cycle metabolites in the urine [33]. In contrast, overexpression of PEPCK could lead to phenotypes such as obesity, lipodystrophy, fatty liver, and T2D [32]. The mechanisms underlying these phenotypes are not yet fully understood, and the same applies to the rs4810083 variant. Depending on the variant’s effect on PEPCK expression, it is possible that rs4810083 (T/T) may reduce PEPCK expression, leading to decreased gluconeogenesis. In such cases, individuals would already have a dysregulated metabolic pathway that metformin could not further mitigate, resulting in nonresponse to the medication. Furthermore, if rs4810083 (T/T) activates PEPCK expression and the gluconeogenesis pathway, it may lead to persistent hyperglycemia despite metformin treatment, as gluconeogenesis remains active. Functional studies are necessary to elucidate the cellular role of *PCK1-*rs4810083 in gluconeogenesis, T2D, and metformin response.

Out of the 9 variants evaluated for metformin safety, two of them, rs316019 and 316009, in the same gene *SLC22A2* were associated with presence of ADRs related to metformin, and no homozygotes for the reference allele (A/A) was observed. These two variants are in perfect linkage disequilibrium, which was observed in this cohort with a genotype pattern identical between both variants. Participants who were homozygous for the alternative allele for rs316019 (C/C) or rs316009 (C/C) reported more ADRs, indicating that being homozygous for rs316019 (C/C) or rs316009 (C/C) increases the odds of having ADRs by 7.33 times. Mixed results were reported regarding the impact of these genotypes on response phenotypes and safety profile related to metformin use. Some findings and associations have reported the impact of the variants on metformin efficacy and not ADRs. Abrahams-October et al. found that the genotype for rs316009 (C/T) may significantly improve metformin response compared to (C/C). The same trend was observed for the rs316019 with the genotype (C/A) but was not significant after correction [34]. On the other hand, regarding pharmacokinetics, Chen and colleagues found in healthy individuals that rs316019 heterozygous (C/A) have a greater renal clearance of metformin than homozygous for the alternative allele (C) [35]. Some research groups have supported this statement, while others concluded the opposite [36,37].

Both variants are part of the *SLC22A*2 gene, which encodes for the organic cation transporter 2 (OCT2), responsible for transporting organic cations, such as metformin, from the bloodstream into the renal tubular cells, primarily in the proximal tubules of the nephron [38]. The most well-known variant in *SLC22A2* is the associated rs316019, a missense mutation that results in the change of an alanine for a serine at position 270 of the protein [39]. This modification appears to impact the transporter’s affinity for metformin and its capacity to clear metformin from the bloodstream into the renal tubule, resulting in an elevated metformin plasma concentration [10]. The other *SLC22A2-*associated variant found, rs316009, is located in a transcription factor binding site [40]. No functional study has been conducted to understand the effects of this variant on OCT2 expression and function. However, due to its location, it is possible that this variant lowers the expression of the transporter by limiting access to the transcription factor binding site, resulting in lower excretion of metformin and plasma accumulation, which could explain the increase in ADRs for the individuals homozygous for the alternative allele (C) [34,41]. An increase in the serum concentration of metformin resulting from a decrease in renal excretion could prolong metformin’s effect, such as inhibiting neoglucogenesis and increasing lactate production, which could increase the possibility of ADRs such as lactic acidosis [37]. In summary, regarding safety, *SLC22A2-*rs316019 and *SLC22A2-*316009 may exert a protective effect against ADRs when individuals are heterozygote. Both *SLC22A2*-associated variants may influence the expression and conformation of the transporters, resulting in lower expression of the transporter and a decrease in its affinity to metformin, therefore limiting its elimination. Such impacts may be responsible for the higher proportion of ADRs observed in individuals homozygous for the alternative allele of both variants, but the exact mechanisms of the functional consequences remain to be further investigated. OCTs play a critical role in metformin’s pharmacokinetics, and accurately characterizing variants in these transporters could help predict ADRs in individuals using metformin for T2D.

To better understand the factors involved in metformin response variability and to characterize the participants, we also evaluated co-variables that could influence metformin efficacy and safety [42] . Associations were found, such as the impact of musculoskeletal, neurological, and gastrointestinal disease on metformin efficacy, and diet, physical activity and mental health issues on its safety profile, but further research is needed. These co-variables, which may modify metformin response, were not included in the genetic analyses as it was not the aim of the study.

A notable finding and strength of this study is the association found between variants and two phenotypes of metformin response evaluated on each cohort’s participant, literature reporting either one or the other. Interviews with participants during visits also provided valuable insights into the complexity of the disease and co-variables influencing metformin response, rarely available in reported studies. Nonetheless, reliance on self-reported dietary habits and lifestyle factors may introduce biases and inaccuracies, which present a limitation. Additionally, certain variables required probing participants’ memory particularly concerning information regarding metformin’s initiation. To control this recall bias, participants were asked to assess their memory reliability when providing information, which helped in evaluating the reliability of the reported information. Also, data collected in medical and pharmacological records served to corroborate the information given by participants and to collect exact lab results to define efficacy phenotypes. Another study limitation is the relatively small size of the cohort for a pharmacogenetics study investigating multiple genetic variants. This cohort size limits the statistical power of the analyses, thereby constraining the ability to detect smaller effect sizes and reducing the generalizability of the findings to a broader population. In addition, the homogeneity of the study population, which is predominantly of European descent, further limits the generalizability of the finding to more diverse populations. To address the small sample size, we controlled by a stringent and targeted candidate variants selection and deep and well performed phenotyping allowing us to find significant associations for two important genes. These findings replicate some associations previously observed in literature, but not all. Further genetic and functional studies with larger and more genetically diverse cohorts are warranted to elucidate the PGx markers of metformin response that would could contribute to optimizing T2D treatment.

## Conclusion

This study reports significant associations between *PCK1* and *SLC22A2* candidate PGx variants and metformin response phenotypes in the treatment of T2D. Additional genetic and functional studies are necessary to elucidate the impact of the associated variants in metformin’s pharmacological and biological mechanisms. Understanding the PGx determinants of metformin response is crucial not only for elucidating its mechanism but also for providing physicians with tools to treat patients more effectively while reducing healthcare costs associated with prescribing inefficient medication. In fact, PGx holds the promise to help optimize patients’ glycemic control and avoid ADRs, thereby reducing their risk of disease progression and occurrence of long-term complications limiting their quality of life.

## Methods

### Study population

This observational retrospective, multicentric, descriptive and analytical case-control pilot study was conducted at “Centre intégré universitaire de santé et de services sociaux du Saguenay–Lac-Saint-Jean” (CIUSSS-SLSJ) research center and at “Centre de recherche du centre hospitalier universitaire de Sherbrooke” (CRCHUS). Adult volunteers included in this study were adults and volunteers. They had a diagnosis of T2D confirmed by a HbA_1C_ between 6.5 and 8.5% at diagnosis. The participants were metformin users for at least one year or used metformin in the last 10 years. All participants allowed access to medical and pharmacological records. Ethical approval was obtained from the Research ethics Committee of the CIUSSS-SLSJ (#2019-045, MP-25-2020-229) and from the Research Ethics Committee of CRCHUS (#MEO-25-2023-4759). The consent obtain was written.

### Recruitment process

Participants were referred by treating physicians or recruited through advertising leaflets distributed in medical clinics, pharmacies, or on social media. Volunteers were contacted to explain the research project and confirm their willingness to participate by delegated team members (SSA, LT, JH). When interested, they were invited to come to one of the recruiting sites for a one-hour visit. Those for whom moving was a limiting factor, a phone appointment was conducted (n =  98). Upon obtaining free and signed informed consent, data collection and sampling of biospecimens (either 10 mL of blood (n =  101) or 4 ml of saliva (n =  4)) were performed and integrated to the principal investigator’s (KT) biobank for future research purposes.

**Fig 1:**
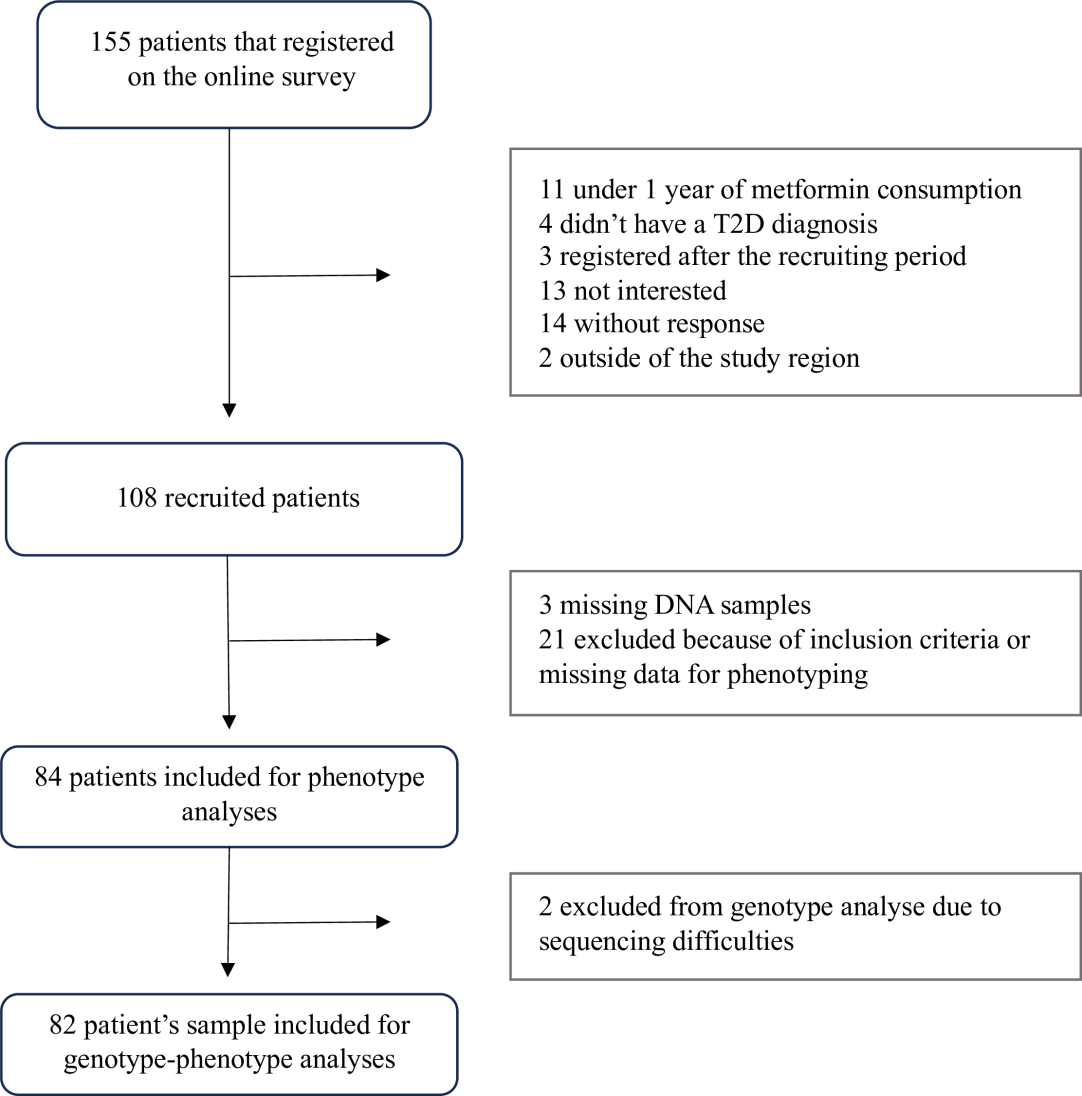
Trajectory and criteria of the participants’ selection. 155 patients were contacted by the research team and 108 patients were recruited. Our of the 108 participants, 3 did not give DNA samples du to lost to follow up. Following medical chart review, it was found that 21 of the recruited patients had either missing data to properly phenotype or didn’t meet the criteria of glycemic value at metformin initiation and they were therefore excluded from analyses. Out of the 84 patients of gave DNA samples, two participants were excluded from genotype-phenotype analyses du to sequencing difficulties. Therefore, 82 participants were included for the genotype-phenotype analyses.

A total of 155 men and women were contacted by the research team, and all were provided with project details and criteria validation. Among them, 108 were found eligible and 105 provided DNA samples. Out of these 105 participants, 21 were excluded from genetic analyses for not meeting the inclusion criteria (having HbA_1C_ under 8,5% at metformin initiation or starting therapy with only metformin) after medical records verification. Fig 1 summarises the study participants selection flowchart.

### Data collection

During their visit, participant self-reported information about demographics, metformin, lifestyle habits, other comorbidities, compliance with dosage instruction, or the presence of ADRs to metformin. The diet was assessed through a self-reported diet score ranging from 1 to 10 established by our research team. Participants were asked to assign themselves a score based on their perception of their diet management at their T2D diagnosis and at study inclusion. Physical activity was also reported by participants and recorded at the same two timepoints. Participants were considered active if they engaged in at least 150 minutes of moderate activity or 75 minutes of intense activity per week, following the World Health Organization (WHO) criteria [22]. All self-reported data was assessed for participant’s memory and data accuracy. To ensure data quality, participants were asked to evaluate their memory, ranging from “I perfectly remember” to “I don’t remember at all”. If the participant didn’t remember the information perfectly, the data was excluded from the analysis. Laboratory data (HbA_1C_, fasting glucose, insulin, creatinine, and the estimated glomerular filtration rate (eGFR, calculated using the CKD-EPI equation)) was collected by reviewing participants’ medical records a posteriori [10]. Ten percent of the laboratory data entry were validated by independent team members (JH, LT) to ensure data quality (discrepancies found were lower than 4%). eGFR after the initiation of metformin was collected to assess dosage changes related to renal impairment. ADRs were mostly self-reported but some were verified in the medical chart if available. Participants also granted access to their archived pharmacological records through a request sent to their pharmacy to collect the concomitant medications at the beginning of metformin treatment. Study data were collected and managed using REDCap electronic data capture tools hosted at Université de Sherbrooke [24,25].

### Phenotyping

The data collected was used to define each participant’s response phenotypes related to efficacy (responder (R) or NR) and safety (ADRs or absence of ADR). The characterization of metformin efficacy involved assessing glycemic control based on serum blood glucose markers that either did or did not meet the targets set by the Canadian consensus on the treatment of T2D in the latest revised version (2018) [26]. To ensure accuracy, the endocrinologist (PP) validated the criteria used to characterize the phenotypes. ADRs were self-reported by participants and, when available, by their treating physician or nurse. The main biomarker targeted was HbA_1C_, along with fasting glucose (8 hours) when available. In instances of discrepancies between these two biomarkers, HbA_1C_ was prioritized.

In this study, response to metformin was defined as achieving adequate blood glucose control after 1 year of treatment with metformin. A responder was defined as having an HbA_1C_ equal to or less than 7% (6.5% being rarely achieved, which would be too restrictive as a criterion) or fasting glucose equal to or below 7 mmol/L after 1 year of treatment with metformin only. On the other hand, a participant was classified as a NR if HbA_1C_ was higher than 7% or fasting glucose levels exceeded 7 mmol/L. Among other parameters used to define a NR, metformin discontinuation during the first year of therapy due to inefficacy or the presence of serious ADRs served as a discriminating factor. Additionally, receiving a second or a third hypoglycemic medication within the first year was considered indicative of non-response.

### Selection of PGx variants

A literature review was conducted on PubMed^®^ and PharmGKB [43] to produce a list of PGx variants reported to be associated with metformin efficacy and ADRs (n =  73, S3 Table). Among them, 34 were selected for genotyping based on their reported minor allelic frequency (greater than 5% on NCBI - presented in Table 3). Variants were excluded if they lacked a locus or if they were triallelic. Some variants were also excluded from genotyping due to their localization in repeated regions or due to the presence of many neighboring variants, which posed a high risk of unsuccessful genotyping.

**Table 3:** Candidate variants for metformin response in T2D treatment and in T2D disease.

Gene Symbol [GeneID] Locus	Variants - SNV^a^	Alleles (REF>ALT)^b^	Allele frequency [allele]^c^	SNV type [amino acids]	Phenotype of response	Ethnicity (Number)
ABCC8 [6833] 11p15.1	rs4148609	C > T	0.34 [T]	Intron	Efficacy	N/A
CAPN10 [11132] 2q37.3	rs3792269	A > G	0.14 [G]	Synonymous [Pre200Pre]	Efficacy	N/A (144)
CDKN2 [1029] 9p21.3	rs10811661	T > C	0.16 [C]	N/A	Efficacy	N/A
CPA6 [57094] 8q13.2	rs2162145	T > C	0.41 [C]	2KB Upstream	Efficacy	CAUC (845)
CSMD1 [64478] 8p23.2	rs2954625	C > T	0.27 [T]	Intron	Efficacy	MG (1056)
GCK [2645] 7p13	rs2908289	G > A	0.21 [A]	Intron	Efficacy	N/A
HFN1B [6928] 17q12	rs11868513	G > A	0.16 [A]	Intron	Efficacy	N/A (927)
HNFF4A [3172] 20q13.12	rs11086926	T > G	0.13 [G]	3’UTR	Efficacy	MG (927)
IRS1 [3667] 2q36.3	rs1801278	C > T	0.05 [T]	Missense [Gly972Arg]	Efficacy	N/A
KCNJ11 [3767] 11p15.1	rs5219	T > C	0.36 [C]	Missense	Efficacy	CAUC (317)
	rs7124355	A > G	0.26 [G]	N/A	Efficacy	N/A
MEF2A [4205] 15q26.3	rs424892	C > T	0.25 [T]	Intron	Efficacy	N/A
NBEA [26960] 13q13.3	rs57081354	T > C	0.08 [C]	Intron	Efficacy	MG (1312)
PCK1 [5105] 20q13.31	rs4810083	T > C	0.45 [C]	2KB Upstream	Efficacy	N/A (148)
PPARGC1A [10891] 4p15.2	rs10213440	T > C	0.21 [C]	Intron	Efficacy	N/A (148)
PPARGC1B [133522] 5q32	rs741579	A > G	0.25 [G]	N/A	Efficacy	N/A
PRKAA1 [5562] 5p13.1	rs249429	C > T	0.29 [T]	Intron	Efficacy	N/A (144)
PRKAA2 [108079] 1p32.2	r9803799	T > G	0.11 [G]	Non-coding	Efficacy	N/A
SLC22A1 [6580] 6q25.3	rs594709	G > A	0.35 [A]	Intron	Safety	Asian (53)
	rs1867351	T > C	0.25 [C]	Synonymous [Ser52Ser]	Safety	MG (106)
	rs12208357	C > T	0.05 [T]	Missense [Arg61Cys]	Safety	MG (106); unknown (12); CAUC (208) (4557)
SLC22A2 [6582] 6q25.3	rs316019	A > C	0.10 [C]	Missense [Ala270Ser]	Safety	MG (106) (23) European (50)(5224)(148); Asian (96); N/A (34)
	rs3119309	C > T	0.09 [T]	Intron	Safety	N/A
	rs316009	T > C	0.09 [C]	Intron	Safety	MG (1056)
SLC22A3 [6581] 6q25.3	rs2076828	C > G	0.43 [G]	Non-coding	Safety	N/A (57)
SLC2A2 [20526] 3q26.2	rs8192675	T > C	0.42 [C]	Intron	Safety	CAUC (10577)
SLC47A1 [55244] 17p11.2	rs8065082	C > T	0.43 [T]	Intron	Safety	MG (990)
	rs2252281	T > C	0.37 [C]	5’ UTR	Safety	CAUC (2651) (50); MG (57) (145) (106); N/A (34)
	rs2289669	G > A	0.32 [A]	Intron	Safety	Asian (220) (53); CAUC (148) (331) (5205) (50); N/A (34); MG (23) (106)
SLC47A2 [146802] 17p11.2	rs12943590	G > A	0.28 [A]	Intron	Safety	Asian (98) (12)(12)(96); CAUC (189) (40); MG (32) (57) (106); N/A (34)
	rs34834489	G > A	0.27 [A]	Upstream gene	Safety	Asian (12)
SP1 [6667] 12q13.13	rs784888	G > C	0.11 [C]	Intron	Safety	MG (440) (57) (106)
STK11 [6794] 19p13.3	rs741765	C > T	0.24 [T]	Intron	Efficacy	N/A
TCF7L2 [6934] 10q25.2-q25.3	rs7903146	C > T	0.27 [T]	Intron	Efficacy	MG (608)

Abbreviations: CAUC, Caucasian; HbA_1C_, glycated hemoglobin; MG, multiple group; N/A, not available or unknown.

^a^Data from GRCh38/hg38 version; http://ncbi.nlm.nih.gov/snp/.

^b^The reference allele and the alternative allele are based on the National Center of Biotechnology Information (NCBI) classification.

^c^From ALFA project.

### Genotyping

Among the 108 participants, 105 provided biospecimens for DNA genotyping. Blood aliquots were prepared for DNA isolation using Qiagen Puregene kit (Hilden, Germany) following standardized laboratory procedures for genetic analysis. Saliva sampling was conducted using the GenoTech^®^ saliva sample collection kit OG-500 (Ontario, Canada) and sent through postal mail collection according to the manufacturer’ instructions. DNA was extracted from saliva using the reagent prepIT.L2P from DNA GenoTech^®^ (Ontario, Canada). The genotyping technology utilized was the Mass Array System iPlex Assay multiplex genotyping, conducted on the Agena Bioscience platform (San Diego, USA). The Genome Québec “Centre d’expertises et de services” (CES) (Montréal, Canada) is equipped with this platform and operates following standardized laboratory procedures. DNA samples were shipped after isolation according to CES quality standards.

### Statistical analyses

Descriptive analyses were conducted to characterize the cohort. For dichotomous and categorical variables, Fisher’s exact tests were used to compared responders to NRs and presence or absence of ADRs. For continuous variables, the Wilcoxon rank-sum test was used for comparison between groups (responders vs NR and presence vs absence of ADRs). Potential relationships (p < 0.05) were evaluated with *post hoc* pairwise 2x2 Fisher tests, followed by Holm-Bonferroni correction. A Hardy-Weinberg equilibrium exact test was performed on each genetic variant. Variants were classified for their role in metformin mechanism. All variants regarding metformin pharmacodynamics and T2D development were put together and analyzed for the efficacy phenotypes of metformin. On the other hand, if a gene had a variant that was responsible for metformin pharmacokinetics, such as transporters, this gene was automatically analyzed for the safety profile of metformin. If a variant was previously associated with efficacy and safety to metformin, safety was prioritized. This resulted in 14 variants analysed for phenotype response and 9 variants analyzed for safety. Three genetics models were tested for each variant: codominant (AA vs AB vs BB), dominant (AA vs. AB/BB), and recessive (AA/AB vs. BB). Multiple testing corrections were performed according to the method proposed by Li, J. & Ji, L. (2005) for adjusting multilocus analyses by calculating the effective number of variants analyzed [28]. Specifically, Bonferroni correction for genetic analyses of metformin’s efficacy was conducted for an effective number of 13 variants, and Bonferroni correction for metformin’s safety was performed for an effective number of 8 variants. If the p-value after correction was < 0.05, the result was considered significant. For significant associations (adjusted p-value < 0.05), a logistic regression model was conducted to quantify the effect by an odds ratio along with a Wald 95% confidence interval.

## Supporting information

S1 Table Characteristics of the participants. (DOCX)

S2 Table Genotype distribution of candidate variants for metformin response in T2D treatment. (DOCX)

S3 Table Gene related to response to metformin in type 2 diabetes treatment and type 2 diabetes disease. (DOCX)

S4 Table Genotype distribution for the dominant model between metformin’s efficacy phenotypes in the studied cohort. (DOCX)

S5 Table Genotype distribution for the dominant model between metformin’s safety phenotypes in the studied cohort. (DOCX)

## Abbreviations

ADR: Adverse drug reaction
ATM: Ataxia-telangiectasia
BMI: Body mass index
CIUSSS-SLSJ: “Centre intégré universitaire de santé et de services sociaux du Saguenay–Lac-Saint-Jean”
CRCHUS: “Centre de recherche du centre hospitalier universitaire de Sherbrooke”
eGFR: Estimated glomerular filtration rate
GLUT2: Glucose transporter member 2
HbA1C: Glycated hemoglobin
HWE: Hardy Weinberg Equilibrium
MAF: Minor allele frequency
NR: Non-responder
OCT: Organic Cation Transporter
PCK1: Phosphoenolpyruvate carboxykinae gene
PEPCK: Phosphoenolpyruvate carboxykinae enzyme
PGx: Pharmacogenetics
SLC: Solute Carrier family
SNV: , Single-nucleotide variants
T1D: Type 1 diabetes
T2D: Type 2 diabetes
