# Supplementary material for: PCK1 and SLC22A2 gene variants associated with response to metformin treatment in type 2 diabetes
Source: PLoS One. 2025 Feb 10;20(2):e0305511. doi: 10.1371/journal.pone.0305511 (PMC11809887; doi:10.1371/journal.pone.0305511)
Supplement: S1 Table — (DOCX) [file pone.0305511.s001.docx]

# ***PCK1* and *SLC22A2* gene variants associated with response to metformin treatment in type 2 diabetes**

**Authors:**

Sophie St-Amour, Laurence Tessier, Janie Harnois, Catherine Allard, Alexandre Lavoie, Philippe Caron, Luigi Bouchard, Patrice Perron, Karine Tremblay

# **Supplementary material**

**Characteristics of the participants**

Of all the participants, 53.6% (n = 45) were women, and a majority were of European descent 98.8%). The mean age at T2D diagnosis was 56.0 (±10.2) years, and at study inclusion, was 65.1 (±11.2) years. The mean BMI at diagnosis was 31.8 (±6.2) kg/$m^{2}$, and 31.0 (±6.3) kg/$m^{2}$ at study inclusion, which are both considered as obese individuals according to the Canadian Guidelines [32]. Regarding lifestyle habits, participants reported a diet score at T2D diagnosis of 5.0/10 [1.0-8.0] and a higher diet score at study inclusion 6.0/10 [3.2-8.0]. Participants also reported an increased physical activity adherence, from 28.9% (n = 24) participants were active at T2D diagnosis to 38.1% (n = 32) participants at study inclusion, as per WHO guidelines [33]. At T2D diagnosis, most participants had moderate alcohol use 75% (n = 63), 48.8% (n = 41) were former tobacco users, and 90.5% (n = 76) participants had never taken other any drugs. Common comorbidities included metabolic disorders other than T2D (83.3%), cardiovascular diseases (71.4%), and musculoskeletal diseases (65.5%).

Twenty-five percent (n = 21) of the participants were NRs, whereas 59.0% (n = 49) of the participants reported experiencing at least one ADR, diarrhea being the most reported. Two participants were excluded for toxicity related to metformin, for safety analysis because they didn’t remember if they had any ADR. The median metformin dosage at initiation of metformin was 1000 [500-1000] mg per day and 1 year after its initiation was 1000 [1000-1700] mg per day. At study inclusion, 93.9% (n = 77) participants were still treated with metformin, while 68.4% (n = 54) of them were also taking at least one other antidiabetic medication.

**Co-variables associated with response to metformin in T2D**

All variables presented in supplementary Table S1 were tested for potential association with metformin response in T2D patients to characterize participants. Among all tested variables, six were significantly associated with response phenotypes. Two out of six variables were self-reported comorbidities at study inclusion. The presence of musculoskeletal diseases was associated with metformin responders (74.6%) compared to NRs (38.1%) (p-value = 0.004) as well as gastrointestinal diseases, which are more frequent in responders (65.1%) compared to NRs (33.3%) (p-value = 0.021). No specific disease reported among these two classes of comorbidities was significantly associated with response phenotypes (data not shown). As expected, based on the phenotype criteria, the levels of HbA_1C_ and fasting glucose one year after metformin initiation showed a significant difference (p-value = <0.0001). One year after metformin initiation, responders levels of HbA_1C_ were at 6.3% (±0.4) and fasting glucose was at 6.4% (±0.8) mmol/L compared to those of NRs which were at 8.0% (±1.2) and 8.0% (±1.2) mmol/L. Additionally, there was also a disparity in HbA_1C_ at the initiation of metformin treatment (p-value = 0.041), with NRs exhibiting higher HbA_1C_ levels of 8.4 (±2.5) mmol/L compared to responders levels of 7.2 (±1.2) mmol/L. Finally, taking another antidiabetic drug at study inclusion was also associated with metformin responses (p-value = 0.013), where 60.3% (35) of responders were taking another antidiabetic drug compared to 90.5% (19) of NRs. Safety was not associated with metformin responses and metformin dosage at initiation and 1 year after its initiation was comparable between responders and NRs (supplementary Table S1).

**Co-variables associated with safety to metformin in T2D**

The same variables were also tested for possible impact on metformin ADRs occurrence, regardless of efficacy response phenotype, to characterize participants (supplementary Table S1). The first three variables associated with ADRs pertain to lifestyle variables. Diet scores reported by participants at T2D diagnosis and at study inclusion were associated with ADR occurrence (p-values = 0.033 and 0.004, respectively). Individuals without ADR had a higher median score of 6.0/10 [1.6-8.0] compared to participants presenting ADRs, who had a median score of 4.0/10 [1.0-8.0] at T2D diagnosis. The same trend was observed for diet score at study inclusion (7.0/10 [5.0-8.0] for individuals without ADR compared to 6.0/10 [2.0-8.0] for those with ADRs). Reported physical activity at study inclusion was also associated with presence of ADRs (p-value = 0.041) and these individuals were less active (28.6% active) than those without ADR (51.5% active). The two other variables associated with toxic effect of metformin are “mental health issues” (p-value = 0.011), where individuals with ADRs (49.0%) reported more mental health issues compared to those without ADR (21.2%), and “other medical conditions” (p-value = 0.016), where individuals with ADRs (20,4%) reported less other medical conditions than those without ADR (48.5%). However, no specific disease for these two categories of comorbidities was significantly associated (data not shown).

**Table S1. Characteristics of the participants**

|  | | | | |  | **Total**  **(n = 84)** | **Efficacy**  **(n = 84)** | | |  | **Safety**  **(n = 82)** | | |
| --- | --- | --- | --- | --- | --- | --- | --- | --- | --- | --- | --- | --- | --- |
|  |  |  |  |  |  |  | **R**  **(n = 63)** | **NR**  **(n = 21)** | **p-valu**$e^{a}$ |  | **ADR**$\mathbf{s}^{b}$  **(n = 49)** | **No-ADR**  **(n = 33)** | **p-value** |
| ***Demographic parameter***$\boldsymbol{s}^{\boldsymbol{c}}$ | | | | |  |  |  |  |  |  |  |  |  |
| Age at study inclusion,  mean years (SD) | | | | |  | 65.1 (11.2) | 65.6 (11.1) | 63.5 (11.9) | 0.460 |  | 63.3 (11.9) | 67.8 (10.2) | 0.285 |
| Age at diagnosis,  mean years (SD) | | | | |  | 56.0 (10.2) | 56.9 (10.4) | 53.1 (9.4) | 0.143 |  | 54.6 (10.8) | 58.4 (9.2) | 0.462 |
| Sex, *n* (%) |  |  |  |  |  |  |  |  |  |  |  |  |  |
| Female |  |  |  |  |  | 45 (53.6) | 36 (57.1) | 9 (42.9) | 0.316 |  | 29 (59.2) | 15 (45.5) | 0.263 |
| Male |  |  |  |  |  | 39 (46.4) | 27 (42.9) | 12 (57.1) |  |  | 20 (40.8) | 18 (54.5) |  |
| Ethnicity, *n* (%) | |  |  |  |  |  |  |  |  |  |  |  |  |
| European descent | |  |  |  |  | 83 (98.8) | 62 (98.4) | 21 (100.0) | 1.000 |  | 49 (100.0) | 32 (97.0) | 0.402 |
| African | |  |  |  |  | 1 (1.2) | 1 (1.6) | 0 (0) |  |  | 0 (0) | 1 (3.0) |  |
| Body mass index at study inclusion,  mean kg/m2 (SD) | | | | | 31.0 (6.3) | | 31.3 (6.5) | 30.1 (5.5) | 0.613 |  | 31.8 (6.2) | 29.9 (6.5) | 0.279 |
| Body mass index at diagnosis,  mean kg/m2 (SD) | | | | |  | 31.8 (6.2) | 31.7 (6.5) | 32.0 (5.4) | 0.843 |  | 32.4 (6.4) | 30.9 (6.0) | 0.271 |
| ***Life habits*** |  |  |  |  |  |  |  |  |  |  |  |  |  |
| Alcohol use at study inclusion, *n* (%$)^{d}$ | | | | |  |  |  |  |  |  |  |  |  |
| Excessive | |  |  |  |  | 4 (4.8) | 2 (3.2) | 2 (9.5) | 0.161 |  | 2 (4.1) | 1 (3.0) | 0.844 |
| Moderate | |  |  |  |  | 59 (70.2) | 47 (74.6) | 12 (57.1) |  |  | 35 (71.4) | 23 (69.7) |  |
| Former |  |  |  |  |  | 7 (8.3) | 6 (9.5) | 1 (4.8) |  |  | 3 (6.1) | 4 (12.1) |  |
| Never |  |  |  |  |  | 14 (16.7) | 8 (12.7) | 6 (28.6) |  |  | 9 (18.4) | 5 (15.2) |  |
| Alcohol use at diagnosis, *n* (%) | | | | |  |  |  |  |  |  |  |  |  |
| Excessive | |  |  |  |  | 5 (6.0) | 3 (4.8) | 2 (9.5) | 0.120 |  | 2 (4.1) | 2 (6.1) | 0.859 |
| Moderate | |  |  |  |  | 63 (75.0) | 50 (79.4) | 13 (61.9) |  |  | 36 (73.5) | 26 (78.8) |  |
| Former |  |  |  |  |  | 3 (3.6) | 3 (4.8) | 0 (0) |  |  | 2 (4.1) | 1 (3.0) |  |
| Never |  |  |  |  |  | 13 (15.5) | 7 (11.1) | 6 (28.6) |  |  | 9 (18.4) | 4 (12.1) |  |
| Tobacco use at study inclusion, *n* (%) | | | | |  |  |  |  |  |  |  |  |  |
| Current |  |  |  |  |  | 5 (6.0) | 4 (6.3) | 1 (4.8) | 0.097 |  | 3 (6.1) | 2 (6.1) | 0.812 |
| Former |  |  |  |  |  | 51 (60.7) | 42 (66.7) | 9 (42.9) |  |  | 31 (63.3) | 18 (54.5) |  |
| Never |  |  |  |  |  | 28 (33.3) | 17 (27.0) | 11 (52.4) |  |  | 15 (30.6) | 13 (39.4) |  |
| Tobacco use at diagnosis, *n* (%) | | | | |  |  |  |  |  |  |  |  |  |
| Current |  |  |  |  |  | 15 (17.9) | 12 (19.0) | 3 (14.3) | 0.114 |  | 9 (18.4) | 4 (12.1) | 0.649 |
| Former |  |  |  |  |  | 41 (48.8) | 34 (54.0) | 7 (33.3) |  |  | 25 (51.0) | 16 (48.5) |  |
| Never |  |  |  |  |  | 28 (33.3) | 17 (27.0) | 11 (52.4) |  |  | 15 (30.6) | 13 (39.4) |  |
| Drugs use at study inclusion, *n* (%) | | | | |  |  |  |  |  |  |  |  |  |
| Current |  |  |  |  |  | 2 (2.4) | 1 (1.6) | 1 (4.8) | 0.672 |  | 2 (4.1) | 0 (0) | 0.202 |
| Former |  |  |  |  |  | 8 (9.5) | 6 (9.5) | 2 (9.5) |  |  | 6 (12.2) | 1 (3.0) |  |
| Never |  |  |  |  |  | 74 (88.1) | 56 (88.9) | 18 (85.7) |  |  | 41 (83.7) | 32 (97.0) |  |
| Drugs use at diagnosis, *n* (%) | | | | |  |  |  |  |  |  |  |  |  |
| Current |  |  |  |  |  | 2 (2.4) | 1 (1.6) | 1 (4.8) | 0.485 |  | 1 (2.0) | 0 (0) | 0.393 |
| Former |  |  |  |  |  | 6 (7.1) | 4 (6.3) | 2 (9.5) |  |  | 5 (10.2) | 1 (3.0) |  |
| Never |  |  |  |  |  | 76 (90.5) | 58 (92.1) | 18 (85.7) |  |  | 43 (87.8) | 32 (97.0) |  |
| Diet, median (IC 95%$)^{e}$ | |  |  |  |  |  |  |  |  |  |  |  |  |
| Diet score at study inclusion | | | | |  | 6.0 [3.2-8.0] | 7.0 [4.0-8.0] | 6.0 [2.0-8.0] | 0.103 |  | 6.0 [2.0-8.0] | 7.0 [5.0-8.0] | **0.004** |
| Diet score at diagnosis | | | | |  | 5.0 [1.0-8.0] | 5.0 [1.0-8.0] | 5.0 [1.0-8.0] | 0.686 |  | 4.0 [1.0-8.0] | 6.0 [1.6-8.0] | **0.033** |
| Physical activity, *n* (%$)^{f}$ | |  |  |  |  |  |  |  |  |  |  |  |  |
| Active at study inclusion | | | | |  | 32 (38.1) | 23 (36.5) | 9 (42.9) | 0.614 |  | 14 (28.6) | 17 (51.5) | **0.041** |
| Active at diagnosis | |  |  |  |  | 24 (28.9) | 18 (28.6) | 6 (30.0) | 1.000 |  | 10 (20.4) | 13 (40.6) | 0.077 |
| ***Comorbidities, n (%)*** | |  | | |  |  |  |  |  |  |  |  |  |
| Cardiovascular diseas$e^{g}$ | | | | |  | 60 (71.4) | 47 (74.6) | 13 (61.9) | 0.278 |  | 35 (71.4) | 23 (69.7) | 1.000 |
| Metabolic disorde$r^{h}$ | |  | | |  | 70 (83.3) | 52 (82.5) | 18 (85.7) | 1.000 |  | 39 (79.6) | 29 (87.9) | 0.384 |
| Musculoskeletal diseas$e^{i}$ | | | | |  | 55 (65.5) | 47 (74.6) | 8 (38.1) | **0.004** |  | 33 (67.3) | 21 (63.6) | 0.814 |
| Cancer |  |  | | |  | 24 (28.6) | 20 (31.7) | 4 (19.0) | 0.403 |  | 11 (22.4) | 12 (36.4) | 0.213 |
| Respiratory diseas$e^{j}$ | |  | | |  | 33 (39.3) | 28 (44.4) | 5 (23.8) | 0.124 |  | 24 (49.0) | 9 (27.3) | 0.067 |
| Neurological diseas$e^{k}$ | |  | | |  | 9 (10.7) | 9 (14.3) | 0 (0) | 0.104 |  | 6 (12.2) | 3 (9.1) | 0.734 |
| Gastrointestinal diseas$e^{l}$ | | | | |  | 48 (57.1) | 41 (65.1) | 7 (33.3) | **0.021** |  | 32 (65.3) | 15 (45.5) | 0.111 |
| Hepatic diseas$e^{m}$ | |  | | |  | 15 (17.9) | 9 (14.3) | 6 (28.6) | 0.188 |  | 8 (16.3) | 7 (21.2) | 0.576 |
| Renal diseas$e^{n}$ | |  | | |  | 30 (35.7) | 24 (38.1) | 6 (28.6) | 0.600 |  | 15 (30.6) | 13 (39.4) | 0.480 |
| Mental health issue$s^{o}$ | |  | | |  | 32 (38.1) | 26 (41.3) | 6 (28.6) | 0.437 |  | 24 (49.0) | 7 (21.2) | **0.012** |
| Allergies | |  | | |  | 48 (57.1) | 34 (54.0) | 14 (66.7) | 0.446 |  | 31 (63.3) | 17 (51.5) | 0.362 |
| Other medical condition$s^{q}$ | | | | |  | 26 (31.0) | 18 (28.6) | 8 (38.1) | 0.426 |  | 10 (20.4) | 16 (48.5) | **0.015** |
| ***Treatment history*** | | | |  |  |  |  |  |  |  |  |  |  |
| HbA_1C_ , mean % (SD) | | | |  |  |  |  |  |  |  |  |  |  |
| 1 year after metformin initiation | | |  |  |  | 6.7 (0.9) | 6.3 (0.4) | 8.0 (1.2) | **<0.0001** |  | 6.6 (1.0) | 6.8 (0.9) | 0.426 |
| Metformin initiation | | |  |  |  | 7.5 (1.7) | 7.2 (1.2) | 8.4 (2.5) | **0.041** |  | 7.6 (1.7) | 7.2 (1.6) | 0.130 |
| Fasting glucose, mean mmol/L (SD) | | | |  |  |  |  |  |  |  |  |  |  |
| 1 year after metformin initiation | | |  |  |  | 7.1 (1.6) | 6.4 (0.8) | 8.0 (1.2) | **<0.0001** |  | 7.2 (2.0) | 6.9 (1.3) | 0.426 |
| Metformin initiation | | |  |  |  | 8.2 (3.0) | 8.1 (3.1) | 9.3 (1.9) | 0.556 |  | 9.0 (3.8) | 7.1 (1.2) | 0.081 |
| Metformin at study inclusion *n* (%) | | | | |  | 77 (93.9) | 59 (96.7) | 18 (85.7) | 0.103 |  | 43 (91.5) | 32 (97.0) | 0.399 |
| Others antidiabetic drugs at study inclusio$n^{r}$ *n* (%) | | | | |  | 54 (68.4) | 35 (60.3) | 19 (90.5) | **0.013** |  | 31 (68.9) | 21 (65.6) | 0.808 |
| Metformin dosage at initiation, median mg/day (IQR) | | | | |  | 1000 [500-1000] | 1000 [500-1000] | 1500 [1000-1700] | 0.212 |  | 1000 [500-1000] | 1000 [500-1125] | 0.996 |
| Metformin dosage 1 year after initiation, median mg/day (IQR) | | | | |  | 1000 [1000-1700] | 1000 [1000-1700] | 1500 [1000-1000] | 0.510 |  | 1000 [1000-1700] | 1000 [1000-1550] | 0.702 |
| eGFR, mean mL/min/1.73$m^{2}$(SD) | | | |  |  |  |  |  |  |  |  |  |  |
| At study inclusion | | |  |  |  | 81.9 (18.7) | 81.2 (18.6) | 84.0 (19.3) | 0.531 |  | 84.6 (17.6) | 78.6 (18.9) | 0.144 |
| 1 year after metformin initiation | | |  |  |  | 87.0 (16.3) | 85.2 (17.0) | 92.5 (13.1) | 0.184 |  | 88.8 (16.9) | 85.7 (15.3) | 0.577 |
| Metformin initiation | | |  |  |  | 85.1 (16.9) | 83.6 (17.6) | 91.2 (12.8) | 0.150 |  | 85.1 (16.8) | 85.2 (17.5) | 0.823 |
| ***Phenotypes, n (%)*** | |  | | |  |  |  |  |  |  |  |  |  |
| Responders | | | | |  | 63 (75.0) | - | - |  |  | 37 (75.5) | 24 (72.7) | 1.000 |
| Non-responders | | | | |  | 21 (25.0) | - | - |  |  | 12 (24.5) | 9 (27.3) |  |
| With ADRs | | | | |  | 49 (59.8) | 37 (60.7) | 12 (57.1) | 1.000 |  | - | - |  |
| Without ADRs | | | | |  | 33 (40.2) | 24 (39.3) | 9 (42.9) |  |  | - | - |  |

Abbreviations: T2D, type 2 diabetes; R, responders; NR, non-responders; ADR, adverse drug reaction; SD, standard deviation; HbA_1C_ , glycated hemoglobin; eGFR, estimated glomerular filtration rate

1. P-value of Fisher exact test for dichotomous and categorical variables. P-value of Wilcoxon rank-sum test for continuous variables. Bold numbers indicate significance (p-value <0.05).
2. At least one adverse drug reaction self-reported or by primary care physician. ADRs included: diarrhea (n = 29), abdominal pain (n = 15), nausea (n = 7), headache (n = 4), vomiting (n = 2), dizziness (n = 1), gas (n = 1), tiredness (n = 1), constipation (n = 1).
3. Proportion/mean/SD calculated on available data: age at study inclusion (n = 83); age at diagnosis (n = 67); body mass index at diagnosis (n=68); diet score at diagnosis (n = 82); physical activity at diagnosis (n = 83); metformin at study inclusion (n = 82); others antidiabetic drug (n = 79); metformin dosage at initiation (n = 79); metformin dosage 1 year after initiation (n = 78).
4. Based on the recommendations of Quebec government (15 consumptions for men and 12 consumptions for women per week).
5. Self-reported score from 1 to 10 on care given to diet (1 = no attention, 10 = greatest attention).
6. Based on the international world health organization, an adult person is considered active by doing 150 minutes of moderate physical activity or 75 minutes of intense physical activity per week. Physical activity was self-reported.
7. Most common self-reported comorbidities in cardiovascular disease: hypertension (n = 53); arrhythmia (n = 3); heart attack (n = 2).
8. Most common self-reported comorbidities in metabolic disorder: hypercholesterolemia (n = 63); dysthyroid (n = 10); hyperlipidemia (n = 2).
9. Most common self-reported comorbidities in musculoskeletal disease: arthritis/arthroses (n = 38); osteoporosis (n = 3); rheumatoid myositis (n = 1).
10. Most common self-reported comorbidities in respiratory disease: sleep apnea (n = 15); asthma (n = 14); chronic obstructive pulmonary disease (n = 4).
11. Most common self-reported comorbidities in neurological disease: loss of sensitivity in the extremity (n = 6); neuropathy (n = 4); retinopathy (n = 1).
12. Most common self-reported comorbidities in gastrointestinal disease: gastroesophageal reflux (n = 29); constipation (n = 5); irritable bowel syndrome (n = 4).
13. Most common self-reported comorbidities in hepatic disease: gallbladder (n = 4); pancreatitis (n = 2).
14. Most common self-reported comorbidities in renal disease: kidney stone (n = 12); kidney failure (n = 6); urinary tract infection (n = 2).
15. Most common self-reported comorbidities in mental health issues: depression (n = 17); anxiety (n = 10); eating disorder (n = 3).
16. Most common self-reported comorbidities in other medical conditions: Eczema (n = 29); loss of balance (n=7); psoriasis (n = 4).
17. Any other antidiabetic drugs that don’t have metformin.

**Table S2. Genotype distribution of candidate variants for metformin response in T2D treatment**

| **Gene** | **Variant** | **Efficacity** | **Safety** | **Alleles (REF>ALT)** | **Call rate (%)** | **ALTF**  **(n = 82)** | **HMZ REF** | **HTZ** | **HMZ ALT** | **HWE**  **p-value** | **HWE adjusted**  **p-valu**$\mathbf{e}^{a}$ |
| --- | --- | --- | --- | --- | --- | --- | --- | --- | --- | --- | --- |
| *ABCC8* | rs4148609 | Yes | No | C>T | 98.78 | 0.315 | 36 | 39 | 6 | 0.439 | 1.000 |
| *CAPN10* | rs3792269 | Yes | No | A>G | 96.34 | 0.139 | 58 | 20 | 1 | 1.000 | 1.000 |
| *CPA6* | rs2162145 | Yes | No | T>C | 100.00 | 0.805 | 3 | 26 | 53 | 1.000 | 1.000 |
| *CSMD1* | rs2954625 | Yes | No | C>T | 96.34 | 0.165 | 56 | 20 | 3 | 0.425 | 1.000 |
| *GCK* | rs2908289 | Yes | No | G>A | 100.00 | 0.146 | 60 | 20 | 2 | 0.675 | 1.000 |
| *HNF1B* | rs11868513 | Yes | No | G>A | 100.00 | 0.171 | 59 | 18 | 5 | **0.049** | 1.000 |
| *IRS1* | rs1801278 | Yes | No | C>T | 100.00 | 0.110 | 65 | 16 | 1 | 1.000 | 1.000 |
| *KCNJ11* | rs5219 | Yes | No | T>C | 98.78 | 0.562 | 19 | 33 | 29 | 0.119 | 1.000 |
|  | rs7124355 | Yes | No | A>G | 98.78 | 0.593 | 18 | 30 | 33 | **0.039** | 0.936 |
| *NBEA* | rs57081354 | Yes | No | T>C | 100.00 | 0.055 | 73 | 9 | 0 | 1.000 | 1.000 |
| *PCK1* | rs4810083 | Yes | No | T>C | 98.78 | 0.531 | 17 | 42 | 22 | 0.824 | 1.000 |
| *PPARGC1A* | rs10213440 | Yes | No | T>C | 97.56 | 0.188 | 52 | 26 | 2 | 0.726 | 1.000 |
| *SLC22A1* | rs594709 | No | Yes | G>A | 100.00 | 0.648 | 8 | 41 | 32 | 0.464 | 1.000 |
|  | rs1867351 | No | Yes | T>C | 100.00 | 0.250 | 44 | 35 | 3 | 0.374 | 1.000 |
|  | rs12208357 | No | Yes | C>T | 100.00 | 0.037 | 76 | 6 | 0 | 1.000 | 1.000 |
| *SLC22A2* | rs316019 | No | Yes | A>C | 100.00 | 0.902 | 1 | 14 | 67 | 0.555 | 1.000 |
|  | rs316009 | No | Yes | T>C | 100.00 | 0.902 | 1 | 14 | 67 | 0.555 | 1.000 |
| *SLC22A3* | rs2076828 | No | Yes | C>G | 100.00 | 0.415 | 26 | 44 | 12 | 0.494 | 1.000 |
| *SLC47A1* | rs8065082 | No | Yes | C>T | 100.00 | 0.360 | 34 | 37 | 11 | 0.815 | 1.000 |
|  | rs2289669 | No | Yes | G>A | 100.00 | 0.311 | 38 | 37 | 7 | 0.798 | 1.000 |
| *SLC47A2* | rs12943590 | No | Yes | G>A | 96.34 | 0.291 | 39 | 34 | 6 | 0.792 | 1.000 |
|  | rs34834489 | No | Yes | G>A | 93.30 | 0.403 | 28 | 36 | 13 | 0.815 | 1.000 |
| *STK11* | rs741765 | Yes | No | C>T | 98.78 | 0.191 | 52 | 27 | 2 | 0.723 | 1.000 |
| *TCF7L2* | rs7903146 | Yes | No | C>T | 97.56 | 0.388 | 31 | 36 | 13 | 0.642 | 1.000 |
|  |  |  |  |  |  |  |  |  |  |  |  |

Abbreviations : REF, reference allele; ALT, alternative allele; ALTF, alternative allele frequency; HMZ, homozygous; HTZ, heterozygous; HWE, Hardy-Weinberg equilibrium; *ABCC8,* ATP-binding cassette transporter sub-family C member 8; *CAPN10,* Calpain 10; *CPA6, C*arboxypeptidase A6; *CSMD1,* CUB and sushi multiple domains 1; *GCK,* Glucokinase; *HNF1B,* Hepatocyte nuclear factor 1; *IRS1,* Insulin receptor substrate 1; *KCNJ11,* Potassium inwardly rectifying channel subfamily J member 11; *NBEA,* Neurobeachin; *PCK1,* Phosphoenolpyruvate carboxykinae 1; *PPARGC1A,* Peroxisome proliferator-activated receptor gamma coactivator 1-alpha; *SLC22A1,* Solute carrier family 22 member 1; *SLC22A2,* Solute carrier family 22 member 2; *SLC22A3,* Solute carrier family 22 member 3; *SLC47A1,* Solute carrier family 47 member 1; *SLC47A2,* Solute carrier family 47 member 2; *STK11,* Serine/threonine kinase 11; *TCF7L2,* Transcription factor 7-like 2

a. Bold numbers indicate significance of Fisher exact test for Hardy-Weinberg equilibrium with adjusted p-value for multiple testing by Holm-Bonferroni correction.

**Table S3. Gene related to response to metformin in type 2 diabetes treatment and type 2 diabetes disease**

| **Gene**  **Symbol**  **[GeneID]**  **Locus** | **Variants -**  **SNV ^a^** | **Alleles**  **(REF>ALT) ^b^** | **Allele frequency [allele] ^c^** | **SNV type [amino acids]** | **Phenotype of response** | **Ethnicity (Number)** | **Inclusion in analysis** | **ref** |
| --- | --- | --- | --- | --- | --- | --- | --- | --- |
| ABCC8 [6833] 11p15.1 | rs4148609 | C>T | 0.35 [T] | Intron | Efficacity | N/A | Yes | (1) |
| ADIPOR2 [79602] 12p13.33 | rs758027 | T>C | 0.02 [C] | N/A | Efficacity | N/A | No | (2) |
| AMHR2 [269] 12q13.13 | rs784892 | G>A | 0.01 [A] | Intron | MET PK; Efficacity; HbA1c | MG (440) (57) (106) | No | (3), (4) |
| ATM [472] 11q22.3 | rs11212617 | C>A | 0.56 [A] | Intron | Efficacity; PK | N/A (4443); CAUC (1024) (155) (331) Near Eastern (140) MG (2984) | No | (1),(2),(5) |
| CAPN10 [11132] 2q37.3 | rs3792269 | A>G | 0.15 [G] | Synonymous [Pre200Pre] | Efficacity | N/A (144) | Yes | (2),(6) |
| CDKN2 [1029] 9p21.3 | rs10811661 | T>C | 0.17 [C] | N/A | Efficacity | N/A | Yes | (2) |
| CPA6 [57094] 8q13.2 | rs2162145 | T>C | 0.73 [C] | 2KB Upstream | Efficacity | CAUC (845) | Yes | (7) |
| CSMD1 [64478] 8p23.2 | rs2954625 | C>T | 0.21 [T] | Intron | Efficacity | MG (1056) | Yes | (6), (8) |
| EEF1A1P11-RPL7P9 | rs10783050 | T>C | 0.35 [C] | N/A | Efficacity | MG (1056) | No | (9) |
| ENPP1 [5167] 6q23.2 | rs1044498 | A>C, G | 0.17 [C] | Missense [Lys173Gln] | Efficacity | East Asian (107) | No | (2) |
| FMO5 [2330] 1q21.1 | rs7541245 | C>A | 0.03 [A] 0.00 [G] | Intron | Efficacity | N/A (258) | No | (6) |
| GCG [2641] 2q24.2 | rs6733736 | A>G, T | 0.00002 [G] 0.00 [T] | Intron | Efficacity | N/A | No | (2) |
| GCK [2645] 7p13 | rs2908289 | G>A | 0.19 [A] | Intron | Efficacity | N/A | Yes | (2) |
| HFN1B [6928] 17q12 | rs11868513 | G>A | 0.20 [A] | Intron | Efficacity | N/A (927) | Yes | (2) |
| HNFF4A [3172] 20q13.12 | rs11086926 | T>G | 0.10 [G] | 3'UTR | Efficacity | MG (927) | Yes | (2) |
| IL1B 2q14.1 | rs1143623 | C>G | 0.25 [G] | 2KB Upstream | Efficacity HbA1c | Asian (85) | No | (9) |
| IRS1 [3667] 2q36.3 | rs1801278 | C>T | 0.06 [T] | Missense [Gly972Arg] | secondary failure | N/A | Yes | (10) |
| ITLN2 [142683] 1q23.3 | rs6701920 | G>A, C | 0.002 [A] 0.00 [C] | Non-coding | Efficacity | N/A | No | (2) |
| KCNJ11 [3767] 11p15.1 | rs5219 | T>C | 0.65 [C] | Missense | Efficacity | CAUC (317) | Yes | (1), (2), (6) |
|  | rs7124355 | A>G | 0.70 [G] | N/A | Efficacity | N/A | Yes | (2) |
| MEF2A [4205] 15q26.3 | rs424892 | C>T | 0.25 [T] | Intron | Efficacity | N/A | Yes | (2) |
| MEF2D [4209] 1q22 | rs6666307 | T>A, G | 0.002 [A], 0.00 [G] | Intron | Efficacity | N/A | No | (2) |
| N/A | rs578427 | C>T | 0.15 [T] | N/A | MET PK | Asian (12) | No | (11) |
| N/A | rs10747673 | G>A | 0.03 [A] | N/A | Efficacity; HbA1C | MG (440) (57) | No | (6) |
| NBEA [26960] 13q13.3 | rs57081354 | T>C | 0.07 [C] | Intron | Efficacity | MG (1312) | Yes | (6) |
| PCK1 [5105] 20q13.31 | rs4810083 | T>C | 0.54 [C] | 2KB Upstream | Efficacity | N/A (148) | Yes | (2) |
| PPARA [5465] 22q13.31 | rs149711321 | T>C | 0.04 [C] | Intron | MET PK; Efficacity; HbA1c | MG (440) | No | (6) |
|  | rs4253652 | T>C | 0.02 [C] | Intron | Efficacity | N/A | No | (2) |
| PPARGC1A [10891] 4p15.2 | rs10213440 | T>C | 0.18 [C] | Intron | Efficacity | N/A (148) | Yes | (2) |
| PPARGC1B [133522] 5q32 | rs741579 | A>G | 0.05 [G] | N/A | Efficacity | N/A | Yes | (2) |
| PRKAA1 [5562] 5p13.1 | rs249429 | C>T | 0.66 [T] | Intron | Efficacity | N/A (144) | Yes | (2) |
| PRKAA2 [108079] 1p32.2 | r9803799 | T>G | 0.05 [G] | Non-coding | Efficacity | N/A | Yes | (2) |
| PRPF31 [26121] 19q13.42 | rs254271 | C>G | 0.52 [G] | Intron | Efficacity | MG (1312) | No | (6) |
| SLC22A1 [6580] 6q25.3 | rs36056065 (rs35854239) | GTAAGTTG | 0.59382 | Deletion | idem | CAUC (193) | No | (1), (6) |
|  | rs2297374 | C>T | 0.40 [T] | Intron | HbA1c, FPI | N/A | No | (1) |
|  | rs594709 | G>A | 0.63 [A] | Intron | Efficacity | Asian (53) | Yes | (6) |
|  | rs622342 | C>A | 0.65 [A] | Intron | Efficacity | MG (106); CAUC (98) (102) (5434) (50) (140) (98); Asian (122) | No | (1), (2) |
|  | rs1867351 | T>C | 0.21 [C] | Synonymous [Ser52Ser] | MET PK; HbA1c; PPG | MG (106) | Yes | (1) |
|  | rs683369 | G/C/A/T | 0.79 [C] 0.00 [T] | Missense [Leu160Phe] | Efficacity | N/A | No | (2) |
|  | rs12208357 | C>T | 0.06 [T] | Missense [Arg61Cys] | MET PK | MG (106); N/A (12); CAUC (208) (4557) | Yes | (1), (2) |
|  | rs4709400 | C/G/T | 0.12 [G] 0.00 [T] | Intron | FPG, PPG | N/A | No | (1) |
|  | rs2282143 | C>T | 0.02 [T] | Missense | MET PK | Asian (96) | No | (1), (6) |
|  | rs34059508 | G>A | 0.02 [A] | Missense [Gly465Arg] | MET PK | CAUC (50) (208) | No | (1) |
|  | rs34447885 | C>T | 0.001 [T] | Missense [Ser14Phe] | MET PK | N/A | No | (1) |
|  | rs34104736 | C>T | 0.001 [T] | Missense [Ser189Leu] | MET PK | N/A | No | (1) |
|  | rs628031 | A>G, C | 0.61 [G], 0.00 [C] | Missense [Met408Val] | Efficacity, FPG | CAUC (193); Near Eastern (140); Asian (24) | No | (1), (6) |
|  | rs72552763 | ATGAT>AT | 0.13 [AT] | Inframe indel [Met420del] | MET PK | CAUC (4423) (50) (40); N/A (12) (371) | No | (1), (6) |
| SLC22A2 [6582] 6q25.3 | rs316019 | A>C | 0.90 [C] | Missense [Ala270Ser] | MET PK, HbA1c | MG (106) (23) European (50) (5224) (148); Asian (96); N/A (34) | Yes | (1), (2), (6) |
|  | rs3119309 | C>T | 0.10 [T] | Intron | Efficacity | N/A | Yes | (1) |
|  | rs316009 | T>C | 0.90 [C] | Intron | Efficacity, MET PK | MG (1056) | Yes | (8) |
|  | rs662301 | C>T | 0.08 [T] | Non-coding | Efficacity | N/A | No | (2) |
|  | rs145450955 | G>A | 0.00 [A] | Missense [Thr201Met] | MET PK, HbA1c, FPG, HOMA-IR | N/A | No | (1) |
|  | rs201919874 | G>A | 0.00 [A] | Missense [Thr199Ile] | MET PK | N/A | No | (1) |
| SLC22A3 [6581] 6q25.3 | rs2076828 | C>G | 0.43 [G] | Non-coding | Efficacity; glucose AUC after glucose tolerance test | N/A (57) | Yes | (6) |
|  | rs3088442 | G>A | 0.35 [A] | Non-coding | Efficacity | N/A | No | (12) |
|  | rs8187725 | C>T | 0.00 [T] | Missense  [T400I] | MET PK | N/A | No | (6), (13) |
| SLC2A2 [20526] 3q26.2 | rs8192675 | T>C | 0.33 [C] | Intron | Efficacity | CAUC (10577) | Yes | (1), (6) |
| SLC47A1 [55244] 17p11.2 | rs8065082 | C>T | 0.44 [T] | Intron | HbA1c, Efficacity | MG (990) | Yes | (1), (2) |
|  | rs2252281 | T>C | 0.39 [C] | 5' UTR | Efficacity | CAUC (2651)(50); MG (57)(145)(106); N/A (34) | Yes | (6) |
|  | rs2289669 | G>A | 0.40 [A] | Intron | MET PK; Efficacity; HbA1c | Asian (220)(53); CAUC(148)(331)(5205)(50); N/A (34); MG (23)(106) | Yes | (1), (2) |
|  | rs77630697 | G>A | 0.00 [A] | Missense [Gly64Asp] | MET PK | N/A | No | (1) |
|  | rs76645859 | G>A | 0.00 [A] | Missense [Val480Met] | MET PK | N/A | No | (1) |
| SLC47A2 [146802] 17p11.2 | rs12943590 | G>A | 0.27 [A] | Intron | MET PK, Efficacity | Asian (98) (12)(12)(96); CAUC (189) (40); MG(32) (57) (106); N/A (34) | Yes | (6) |
|  | rs34834489 | G>A | 0.32 [A] | Upstream gene | MET PK | Asian (12) | Yes | (1) |
|  | rs34399035 | C>T | 0.01 [T] | Missense [Gly429Argt] | HbA1c | N/A | No | (1) |
|  | rs146901447 | G>A | 0.001 [A] | Missense [Pro162Leu] | MET PK, Efficacity | N/A | No | (1) |
|  | rs373244724 | T>C | 0.00 [C] | Missense [Tyr273Cys] | MET PK | N/A | No | (1) |
| SP1 [6667] 12q13.13 | rs784888 | G>C | 0.05 [C] | Intron | Efficacity; Met PK | MG (440)(57)(106) | Yes | (6), (3), (4) |
|  | rs2683511 | C>T | 0.07 [T] | Intron | Efficacity; HbA1C; MET PK | MG (440)(57) | No | (3) |
| SRR [63826] 12q13.13 | rs391300 | T>C | 0.62 [C] | Intron | PPG; FPG | Asian (44) | No | (2) |
| STK11 [6794] 19p13.3 | rs741765 | C>T | 0.21 [T] | Intron | Efficacity | N/A | Yes | (2) |
| TCF7L2 [6934] 10q25.2-q25.3 | rs7903146 | C>T | 0.29 [T] | Intron | Efficacity | MG (608) | Yes | (14), (6) |
|  | rs6719578 | C>G | 0.01 [G] |  | Efficacity | MG (927) | No | (15) |

Abbreviations: CAUC, Caucasian; HbA_1C_ , glycated hemoglobin; MET, metformin; MG, multiple groups; N/A, not available or unknown; PK, pharmacokinetics

^a^ Data from GRCh38/hg38 version; http://ncbi.nlm.nih.gov/snp/.

^b^ The reference allele and the alternative allele are based on the National Center of Biotechnology Information (NCBI) classification.

^c^ From ALFA project.

**Table S4. Genotype distribution for the dominant model between metformin’s efficacy phenotypes in the studied cohort**

| **Genotypes *n (%)*** |  |  |  | | **R**  **(n = 61)** | | **NR**  **(n = 21)** | | **p-valu**$\mathbf{e}^{a}$ | | **Adjusted p-value** | |
| --- | --- | --- | --- | --- | --- | --- | --- | --- | --- | --- | --- | --- |
| *ABBC8* - rs4148609 | | | |  | |  | |  | |  | |  |
| CC |  |  | | 28 (46.7%) | | 8 (38.1%) | | 0.612 | | 1.000 | |  |
| CT + TT |  |  | | 32 (53.3%) | | 13 (61.9%) | |  | |  | |  |
| *Capn10* - rs3792269 | | | |  | |  | |  | |  | |  |
| AA |  |  | | 43 (74.1%) | | 15 (71.4%) | | 0.782 | | 1.000 | |  |
| AG + GG |  |  | | 15 (25.9%) | | 6 (28.6%) | |  | |  | |  |
| *CPA6* - rs2162145 | | | |  | |  | |  | |  | |  |
| TT |  |  | | 3 (4.9%) | | 0 (0%) | | 0.566 | | 1.000 | |  |
| TC + CC |  |  | | 58 (95.1%) | | 21 (100.0%) | |  | |  | |  |
| *CSMD1* - rs2954625 | | | |  | |  | |  | |  | |  |
| CC |  |  | | 44 (74.6%) | | 12 (60.0%) | | 0.259 | | 1.000 | |  |
| CT + TT |  |  | | 15 (25.4%) | | 8 (40.0%) | |  | |  | |  |
| *GCK* - rs2908289 | | | |  | |  | |  | |  | |  |
| GG |  |  | | 43 (70.5%) | | 17 (81.0%) | | 0.407 | | 1.000 | |  |
| GA + AA |  |  | | 18 (29.5%) | | 4 (19.0%) | |  | |  | |  |
| *HNF1B* - rs11868513 | | | |  | |  | |  | |  | |  |
| GG |  |  | | 43 (70.5%) | | 16 (76.2%) | | 0.780 | | 1.000 | |  |
| GA + AA |  |  | | 18 (29.5%) | | 5 (23.8%) | |  | |  | |  |
| *IRS1* - rs1801278 | | | |  | |  | |  | |  | |  |
| CC |  |  | | 48 (78.7%) | | 17 (81.0%) | | 1.000 | | 1.000 | |  |
| CT + TT |  |  | | 13 (21.3%) | | 4 (19.0%) | |  | |  | |  |
| *KCNJ11* - rs5219 | | | |  | |  | |  | |  | |  |
| TT |  |  | | 14 (23.3%) | | 5 (23.8%) | | 1.000 | | 1.000 | |  |
| CT + CC |  |  | | 46 (76.7%) | | 16 (76.2%) | |  | |  | |  |
| *KCNJ11* - rs7124355 | |  | |  | |  | |  | |  | |  |
| AA |  |  | | 13 (21.7%) | | 5 (23.8%) | | 1.000 | | 1.000 | |  |
| AG + GG |  |  | | 47 (78.3%) | | 16 (76.2%) | |  | |  | |  |
| *NBEA* - rs57081354 | | | |  | |  | |  | |  | |  |
| TT |  |  | | 53 (86.9%) | | 20 (95.2%) | | 0.435 | | 1.000 | |  |
| TC + CC |  |  | | 8 (13.1%) | | 1 (4.8%) | |  | |  | |  |
| *PCK1* - rs4810083 | | | |  | |  | |  | |  | |  |
| TT |  |  | | 15 (24.6%) | | 2 (10.0%) | | 0.216 | | 1.000 | |  |
| TC + CC |  |  | | 46 (75.4%) | | 18 (90.0%) | |  | |  | |  |
| *PPARGC1A* - rs10213440 | | | |  | |  | |  | |  | |  |
| TT |  |  | | 39 (65.0%) | | 13 (65.0%) | | 1.000 | | 1.000 | |  |
| TC + CC |  |  | | 21 (35.0%) | | 7 (35.0%) | |  | |  | |  |
| *STK11* - rs741765 | | | |  | |  | |  | |  | |  |
| CC |  |  | | 35 (58.3%) | | 17 (81.0%) | | 0.071 | | 0.923 | |  |
| CT + TT |  |  | | 25 (41.7%) | | 4 (19.0%) | |  | |  | |  |
| *TCF7L2* - rs2908289 | | | |  | |  | |  | |  | |  |
| CC |  |  | | 24 (40.0%) | | 7 (35.0%) | | 0.794 | | 1.000 | |  |
| CT + TT |  |  | | 36 (60.0%) | | 13 (65.0%) | |  | |  | |  |

Abbreviations : R, responders; NR, non-responders; *ABCC8,* ATP-binding cassette transporter sub-family C member 8; *CAPN10,* Calpain 10; *CPA6, C*arboxypeptidase A6; *CSMD1,* CUB and sushi multiple domains 1; *GCK,* Glucokinase; *HNF1B,* Hepatocyte nuclear factor 1; *IRS1,* Insulin receptor substrate 1; *KCNJ11,* Potassium inwardly rectifying channel subfamily J member 11; *NBEA,* Neurobeachin; *PCK1,* Phosphoenolpyruvate carboxykinase 1; *PPARGC1A,* Peroxisome proliferator-activated receptor gamma coactivator 1-alpha; *STK11,* Serine/threonine kinase 11; *TCF7L2,* Transcription factor 7-like 2.

1. P-value of Fisher exact test comparing Rs to NRs. Bold numbers indicate significance (p<0.05) and asterisks (*) indicate significance for *post-hoc* using Holm–Bonferroni adjustment*.*

**Table S5. Genotype distribution for the dominant model between metformin’s safety phenotypes in the studied cohort**

| **Genotypes *n (%)*** |  |  |  | | **ADRs**  **(n = 47)** | **No-ADR**  **(n = 33)** | **p-valu**$\mathbf{e}^{a}$ | **Adjusted p-value** |  |
| --- | --- | --- | --- | --- | --- | --- | --- | --- | --- |
| *SLC22A1* - rs594709 | | | |  |  |  |  |  |  |
| GG |  |  | |  | 6 (13.0%) | 2 (6.1%) | 0.457 | 1.000 |  |
| GA + AA |  |  | |  | 40 (87.0%) | 31 (93.9%) |  |  |  |
| *SLC22A1* - rs1867351 | | |  | |  |  |  |  |  |
| TT |  |  | |  | 25 (53.2%) | 18 (54.5%) | 1.000 | 1.000 |  |
| TC + CC |  |  | |  | 22 (46.8%) | 15 (45.5%) |  |  |  |
| *SLC22A1* - rs12208357 | | |  | |  |  |  |  |  |
| CC |  |  | |  | 44 (93.6%) | 30 (90.9%) | 0.687 | 1.000 |  |
| CT + TT |  |  | |  | 3 (6.4%) | 3 (9.1%) |  |  |  |
| *SLC22A2* - rs316019/rs316009 | | | |  |  |  |  |  |  |
| AA/TT |  |  | |  | 0 (0%) | 0 (0%) | - | - |  |
| AC/TC + CC/CC |  |  | |  | 47 (100.0%) | 33 (100.0%) |  |  |  |
| *SLC22A3* - rs2076828 | | | |  |  |  |  |  |  |
| CC |  |  | |  | 10 (21.3%) | 16 (48.5%) | **0.015** | 0.120 |  |
| CG + GG |  |  | |  | 37 (78.7%) | 17 (51.5%) |  |  |  |
| *SLC47A1* - rs8065082 | | | |  |  |  |  |  |  |
| CC |  |  | |  | 21 (44.7%) | 12 (36.4%) | 0.497 | 1.000 |  |
| CT + TT |  |  | |  | 26 (55.3%) | 21 (63.6%) |  |  |  |
| *SLC47A1* - rs2289669 | | |  | |  |  |  |  |  |
| GG |  |  | |  | 24 (51.1%) | 13 (39.4%) | 0.365 | 1.000 |  |
| GC + AA |  |  | |  | 23 (48.9%) | 20 (60.6%) |  |  |  |
| *SLC47A2* - rs12943590 | | | |  |  |  |  |  |  |
| GG |  |  | |  | 23 (52.3%) | 15 (45.5%) | 0.647 | 1.000 |  |
| GA + AA |  |  | |  | 21 (47.7%) | 18 (54.5%) |  |  |  |
| *SLC47A2* - rs34834489 | | |  | |  |  |  |  |  |
| GG |  |  | |  | 19 (43.2%) | 9 (29.0%) | 0.236 | 1.000 |  |
| GA + AA |  |  | |  | 25 (56.8%) | 22 (71.0%) |  |  |  |

Abbreviations: ADR, adverse drug reaction; *SLC22A1,* Solute carrier family 22 member 1; *SLC22A2,* Solute carrier family 22 member 2; *SLC22A3,* Solute carrier family 22 member 3; *SLC47A1,* Solute carrier family 47 member 1; *SLC47A2,* Solute carrier family 47 member 2; *STK11,* Serine/threonine kinase 11; *TCF7L2,* Transcription factor 7-like 2

1. P-value of Fisher exact test comparing ADRs to No-ADR. Bold numbers indicate significance (p<0.05) and asterisks (*) indicate significance for *post-hoc* using Holm–Bonferroni adjustment*.*

**References**

1. Mannino GC, Andreozzi F, Sesti G. Pharmacogenetics of type 2 diabetes mellitus, the route toward tailored medicine. Diabetes Metab Res Rev. mars 2019;35(3):e3109.

2. Maruthur NM, Gribble MO, Bennett WL, Bolen S, Wilson LM, Balakrishnan P, et al. The Pharmacogenetics of Type 2 Diabetes: A Systematic Review. Diabetes Care. mars 2014;37(3):876‑86.

3. Goswami S, Yee SW, Stocker S, Mosley JD, Kubo M, Castro R, et al. Genetic variants in transcription factors are associated with the pharmacokinetics and pharmacodynamics of metformin. Clin Pharmacol Ther. sept 2014;96(3):370‑9.

4. Santoro AB, Botton MR, Struchiner CJ, Suarez-Kurtz G. Influence of pharmacogenetic polymorphisms and demographic variables on metformin pharmacokinetics in an admixed Brazilian cohort. Br J Clin Pharmacol. mai 2018;84(5):987‑96.

5. Out M, Becker ML, van Schaik RH, Lehert P, Stehouwer CD, Kooy A. A gene variant near ATM affects the response to metformin and metformin plasma levels: a post hoc analysis of an RCT. Pharmacogenomics. 1 juin 2018;19(8):715‑26.

6. Srinivasan S, Kaur V, Chamarthi B, Littleton KR, Chen L, Manning AK, et al. TCF7L2 Genetic Variation Augments Incretin Resistance and Influences Response to a Sulfonylurea and Metformin: The Study to Understand the Genetics of the Acute Response to Metformin and Glipizide in Humans (SUGAR-MGH). Diabetes Care. mars 2018;41(3):554‑61.

7. Rotroff DM, Yee SW, Zhou K, Marvel SW, Shah HS, Jack JR, et al. Genetic Variants in CPA6 and PRPF31 Are Associated With Variation in Response to Metformin in Individuals With Type 2 Diabetes. Diabetes. juill 2018;67(7):1428‑40.

8. Goswami S, Yee SW, Xu F, Sridhar SB, Mosley JD, Takahashi A, et al. A Longitudinal HbA1c Model Elucidates Genes Linked to Disease Progression on Metformin. Clin Pharmacol Ther. nov 2016;100(5):537‑47.

9. Xiao D, Zhang SM, Li X, Yin JY, Gong WJ, Zheng Y, et al. IL-1B rs1143623 and EEF1A1P11-RPL7P9 rs10783050 polymorphisms affect the glucose-lowing efficacy of metformin in Chinese overweight or obese Type 2 diabetes mellitus patients. Pharmacogenomics. 2015;16(14):1621‑9.

10. Li Q, Qiao Y, Wang C, Zhang G, Zhang X, Xu L. Associations between two single-nucleotide polymorphisms (rs1801278 and rs2943641) of insulin receptor substrate 1 gene and type 2 diabetes susceptibility: a meta-analysis. Endocrine. 1 janv 2016;51(1):52‑62.

11. Chung JY, Cho SK, Kim TH, Kim KH, Jang GH, Kim CO, et al. Functional characterization of MATE2-K genetic variants and their effects on metformin pharmacokinetics. Pharmacogenet Genomics. juill 2013;23(7):365‑73.

12. Moeez S, Riaz S, Masood N, Kanwal N, Arif MA, Niazi R, et al. Evaluation of the rs3088442 G>A SLC22A3 Gene Polymorphism and the Role of microRNA 147 in Groups of Adult Pakistani Populations With Type 2 Diabetes in Response to Metformin. Canadian Journal of Diabetes. 1 mars 2019;43(2):128-135.e3.

13. Chen L, Pawlikowski B, Schlessinger A, More SS, Stryke D, Johns SJ, et al. Role of organic cation transporter 3 (SLC22A3) and its missense variants in the pharmacologic action of metformin. Pharmacogenet Genomics. nov 2010;20(11):687‑99.

14. Dujic T, Bego T, Malenica M, Velija-Asimi Z, Ahlqvist E, Groop L, et al. Effects of TCF7L2 rs7903146 variant on metformin response in patients with type 2 diabetes. Bosn J Basic Med Sci. nov 2019;19(4):368‑74.

15. Billings LK, Jablonski KA, Warner AS, Cheng YC, McAteer JB, Tipton L, et al. Variation in Maturity-Onset Diabetes of the Young Genes Influence Response to Interventions for Diabetes Prevention. J Clin Endocrinol Metab. 27 avr 2017;102(8):2678‑89.
